# Income-Raising Device in the Antipodes

**Published:** 1920-07-31

**Authors:** Norman Haire

**Affiliations:** Hampstead General and North-West London Hospital.


*To the Editor of* The Hospital.

### Income-Raising Device in the Antipodes.

*To the Editor of* The Hospital.

Sir,—In view of the present poverty of most of the public hospitals, some of your readers may be interested to learn of a method of adding to hospital income which is employed in Australia in such large hospitals as the Royal Prince Alfred Hospital, Sydney, the Melbourne Hospital, the Newcastle Hospital, and the Royal Hospital for Women, Paddington.

It was found that the ordinary visiting hours did not meet the convenience of many people who might be prevented by their business from visiting husband, wife, parent or child. As a result the hospital authorities were continually being requested to permit visitors to enter the wards at all hours. A consent aroused a certain amount of jealousy and discontent among other patients, while a refusal might inflict a real hardship in a genuine case. It was, therefore, decided to establish, in addition to the *free* ordinary visiting hours, certain fixed "*Extra Visiting Hours*," during which visitors would be admitted by ticket on contribution of sixpence towards the hospital funds. The extra visiting hours are arranged to suit the convenience of each individual hospital, and depend on the hours at which the honorary staff do their rounds, etc., but an hour each evening is generally found most suitable for staff and visitor alike.

It was feared that the patients and their friends might regard the charge as an imposition, but the point was emphasised that the original visiting hours were preserved unaltered, and that this was an additional privilege to be enjoyed for a small payment. In practice we found that the public welcomed the innovation. The annual income from this source varied in different hospitals from £300 to £500 for every hundred beds.

The Public Health Department contributes towards the upkeep of all public hospitals an amount equal to that collected from voluntary contributions.—I am, yours faithfully,

Norman Haire, M.B., Ch.M. (Sydney).

Hampstead General and North-West London Hospital.
July 20, 1920.

